# A systematic review and meta-analysis of gene therapy with hematopoietic stem and progenitor cells for monogenic disorders

**DOI:** 10.1038/s41467-022-28762-2

**Published:** 2022-03-14

**Authors:** Francesca Tucci, Stefania Galimberti, Luigi Naldini, Maria Grazia Valsecchi, Alessandro Aiuti

**Affiliations:** 1 Pediatric Immunohematology and Bone Marrow Transplantation Unit, IRCCS San Raffaele Scientific Institute, Milan, Italy; 2 San Raffaele Telethon Institute for Gene Therapy (SR-Tiget), IRCCS San Raffaele Scientific Institute, Milan, Italy; 3 Bicocca Bioinformatics Biostatistics and Bioimaging B4 Center, School of Medicine and Surgery, University of Milano — Bicocca, Monza, Italy; 4 Vita-Salute San Raffaele University, Milan, Italy

**Keywords:** Stem cells, Gene therapy, Gene therapy

## Abstract

Ex-vivo gene therapy (GT) with hematopoietic stem and progenitor cells (HSPCs) engineered with integrating vectors is a promising treatment for monogenic diseases, but lack of centralized databases is hampering an overall outcomes assessment. Here we aim to provide a comprehensive assessment of the short and long term safety of HSPC-GT from trials using different vector platforms. We review systematically the literature on HSPC-GT to describe survival, genotoxicity and engraftment of gene corrected cells. From 1995 to 2020, 55 trials for 14 diseases met inclusion criteria and 406 patients with primary immunodeficiencies (55.2%), metabolic diseases (17.0%), haemoglobinopathies (24.4%) and bone marrow failures (3.4%) were treated with gammaretroviral vector (γRV) (29.1%), self-inactivating γRV (2.2%) or lentiviral vectors (LV) (68.7%). The pooled overall incidence rate of death is 0.9 per 100 person-years of observation (PYO) (95% CI = 0.37–2.17). There are 21 genotoxic events out of 1504.02 PYO, which occurred in γRV trials (0.99 events per 100 PYO, 95% CI = 0.18–5.43) for primary immunodeficiencies. Pooled rate of engraftment is 86.7% (95% CI = 67.1–95.5%) for γRV and 98.7% (95% CI = 94.5–99.7%) for LV HSPC-GT (p = 0.005). Our analyses show stable reconstitution of haematopoiesis in most recipients with superior engraftment and safer profile in patients receiving LV-transduced HSPCs.

## Abstract (web summary)

Ex-vivo gene therapy with hematopoietic stem and progenitor cells (HSPCs) is a promising treatment for monogenic diseases. Here the authors report a systematic review and meta-analysis of available evidence assessing clinical outcomes of HSPC gene therapy from clinical trials.

## Introduction

In the past two decades, gene transfer into hematopoietic stem/progenitor cells (HSPCs) has emerged as a promising treatment for several monogenic diseases, including primary immunodeficiencies (PID), metabolic disorders, haemoglobinopathies and bone marrow failures. Autologous HSPC gene therapy (GT), which belongs to the group of medicinal products classified as advanced therapies medicinal product (ATMP)^1^, is designed to overcome the main limitations related to allogeneic HSPC transplantation (HSCT), such as the availability of a compatible donor, the risk of graft versus host disease (GvHD) and need of post-HSCT immunosuppression, while providing comparable or sometime even superior therapeutic benefit. Recently three ATMPs based on HSPC-GT have been approved for the EU market for the treatment of Adenosine Deaminase Severe Combined Immunodeficiency (ADA-SCID), beta thalassemia and metachromatic leukodystrophy (MLD), respectively^2^. Other products are in advanced stage of development in the EU and US.

Integrating viral vectors stably transfer the therapeutic gene into the cromatin of the patients’ own HSPCs collected from the bone marrow or peripheral blood after mobilization. After reinfusion, gene corrected HSPCs undergo self-renewal and transfer an healthy copy of the gene to daughter blood cells. To date, HSPC-GT works primarily through two mechanisms of action. In the case of PID and haemoglobinopathies, expression of the healthy gene reestablishes normal differentiation and/or function of mature cells such as immune cells or red blood cells. For metabolic disorders, myeloid cells are engineered to express supraphysiological levels of the defective enzyme, which allows functional reconstitution of scavenger activity within various tissues and cross-correction of resident non-hematopoietic cells, including in the central nervous system^2^. The first integrating vectors to be employed were derived from gamma-retroviruses (γRV). The limited gene transfer efficiency into HSPCs and the occurrence of adverse events due insertional mutagenesis in γRV trials accelerated the development of self-inactivating lentiviral vectors (LV) as a delivery platform enabling more effective and safe insertion of therapeutic genes into HSPCs.

Several excellent disease specific reviews have been published in this evolving area which, anyhow, report the main results in descriptive manner, without providing cumulative analyses^3,4^. On the other hand, despite the requirement from national and EU regulatory bodies for active monitoring of delayed adverse events, the lack of centralization currently hampers a thorough and comprehensive collection of the long-term safety and efficacy data of HSPC-GT across various studies and platforms.

Here we review in a systematic manner the literature on monogenic diseases in the field of ex-vivo HSPC-GT with the aim to describe survival, genotoxicity and engraftment of gene corrected HSPCs, across vector platforms and diseases, in a large cohort of patients over a period of 25 years. With a robust clinical follow-up, we observe a stable reconstitution of haematopoiesis with gene-corrected cells in most recipients with a safer genotoxic profile in patients receiving lentiviral-transduced HSPCs. This meta-analysis helps providing a comprehensive picture of the current outcomes of these highly innovative treatments with the goal of informing scientific community, regulatory authorities and clinical practice.

## Results

### Studies selection and characteristics

**Fig. 1:**
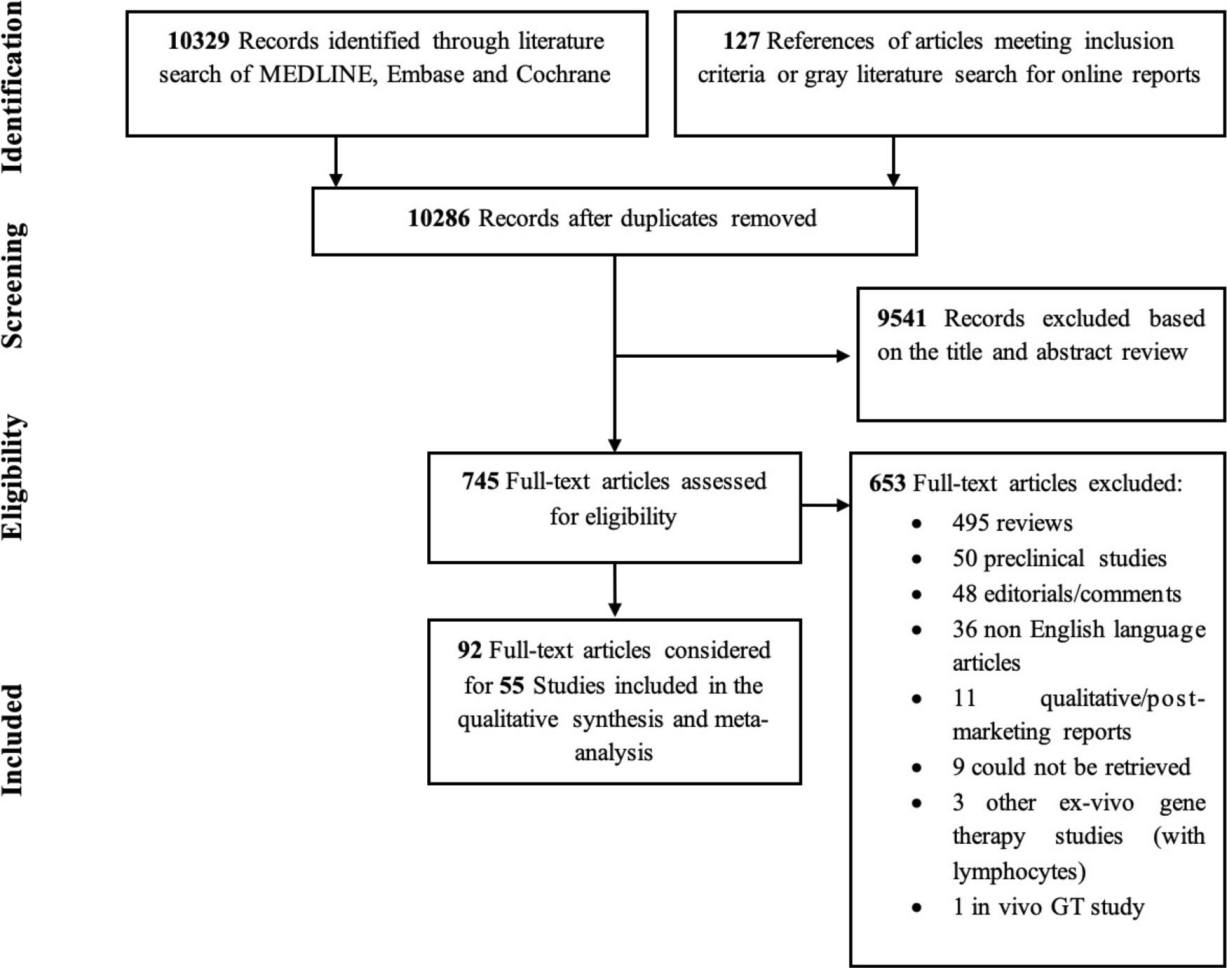
PRISMA diagram detailing the search strategy. PRISMA diagram detailing the search strategy of articles for inclusion in the meta-analysis.

The results obtained from our search strategy are reported through the PRISMA flowchart in Fig. 1. From an initial selection of 10,329 records from literature search and 127 from gray literature that were assessed, 745 records were evaluated as full-texts for eligibility and a total of 55 studies, involving 406 patients, were considered. Overall, none of the studies included in the systematic review showed important methodological flaws as to be excluded from the meta-analysis (Supplementary Text for detailed results; Supplementary Table 1 for data). The selected trials, performed from 1995 to 2020, were all one-arm prospective studies and focused on the treatment of 14 different diseases by ex-vivo HSPC-GT (Table 1). LV was the most often used vector to genetically modify HSPCs [34 trials (61.8%) and 279 patients (68.7%) and a total of 730.6 person-years of observation (PYO)], followed by γRV (20 trials and 118 patients, 36.4% and 29.1%, respectively and a total of 807 PYO) and SIN-γRV (1 trial and 9 patients, 1.8% and 2.2%, respectively, and a total of 18.6 PYO) (Supplementary Table 2). The use of LV was exclusive in trials of metabolic diseases (*n* = 8, 14.5%), Fanconi anemia (FA) (*n* = 3, 5.4%) and hemoglobinopathies (*n* = 11, 20%). In the PID group γRV was more frequently used (*n* = 20, 36.4%) than LV (*n* = 12, 21.8%) or SIN-γRV (*n* = 1, 1.8%). The number of treated patients and the follow-up greatly varied across trials, ranging from one to 29 patients in sample size and 0.5 to 276.58 in total PYO, respectively. Where the conditioning regimen was declared, 21 trials used a non-myeloablative regimen (13 γRV and 8 LV trials), 24 a myeloblative regimen (all LV trials), while no pre-GT conditioning was employed in 8 γRV, 3 LV and 1 SIN-γRV trials (Table 1). Two trials had >one regimen. The median CD34+ cell dose among trials ranged from 0.28 to 23.1 × 10^6^/kg. Collecting all the individual data available, overall, the median CD34+ cell dose infused was 8.95 × 10^6^/kg (range 0.03–71) (260 available individual data points) and median VCN/genome was 1.6 (range 0.05–9.4) (200 available data points).

**Table 1:** Characteristics of the 55 studies included in the meta-analysis.

Publication	Country	CT registration	Disease	N° Patients	Vector	Conditioning	CD34+ *10^6^/kg median (min–max)	VCN copies/genome (min–max)	Follow-up* in months (min–max)
Candotti et al.^38^	USA	NCT00018018	ADA-SCID	10	gRV-ADA	N/S	1.9 (0.7–9.8)	0.1–1.46	30–120
Shaw et al.^39^	USA	NCT00794508	ADA-SCID	10	gRV-ADA	S	6.23 (0.6–8.4)	0.6–2.68	42–84
Aiuti et al.^40–43^	Italy	NCT00598481	ADA-SCID	22	gRV-ADA	S	9.23 (0.9–18.5)	0.3–1.8	12.9–241
Aiuti (PC)§^44^	Italy	Compassionate use	ADA-SCID	2	gRV-ADA	S	17.9 (10.1–25.7)	1.4	28–29
Migliavacca et al.^45^	Italy	NCT03478670	ADA-SCID	12	gRV-ADA	S	11.55 (3.4–19.7)	1.2–2.5	11–43
Otsu et al.^46^	Japan	-	ADA-SCID	2	gRV-ADA	N	1.15 (0.92–1.38)	-	72–120
Gaspar et al.^47^	UK	NCT01279720	ADA-SCID	6	gRV-ADA	S	1.65 (0.5–5.8)	-	24–84
Gaspar et al.^48^	UK	-	ADA-SCID	1	gRV-ADA	S	1.4	-	24
Kohn et al.^49,50^	USA	-	ADA-SCID	3	gRV-ADA	N	-	-	180
Braun et al.^51,52^	Germany	DRKS00000330	WAS	10	gRV-WAS	S	18.25 (2.9–24.9)	1.7–3.2	15–86
Malech et al.^53^	USA	BB IND 6100	X-CGD	5	MFGS RV p47phox	N	2.5 (0.1–4.7)	-	84–120
Siler et al.^54^	Switzerland	NCT00927134	X-CGD	2	gRV-CYBB	S	5.65 (5.3–6)	0.8–1.3	57.6–87
Ott et al.^55^	Germany	NCT00564759	X-CGD	2	gRV-CYBB	S	4.35 (3.6–5.1)	1.5–2.6	27–45
Kang et al.^56^	USA	NCT00394316	X-CGD	3	gRV-CYBB	S	19 (18.9–71)	0.005–4.05	11–36
Kang et al.^57^	Korea	NCT00778882	X-CGD	2	gRV-CYBB	S	5.6 (5.4–5.8)	0.5–2	48
Uchiyama et al.^58^	Japan	-	X-CGD	1	gRV-CYBB	S	6.5	2.63	49
Six; Ginn et al.^59–63^	France; Australia	-	X-SCID	10	gRV-IL-2Rγ	N	5 (1–22)	-	8–180
Gaspar et al.^64^	UK		X-SCID	10	gRV-IL-2Rγ	N	23.1 (6.9–34.1)	-	51–107
Chinen et al.^65^	USA	NCT00028236	X-SCID	3	gRV-IL-2Rγ	N	2.92 (2.85–3.13)	1.1–3.7	12–30
Thrasher et al.^66^	France; USA; UK	-	X-SCID	2	gRV-IL-2Rγ	N	18.9 (2.8–35)	-	6
Gaspar et al.^67^	UK	NCT01380990	ADA-SCID	5	EFS-ADA LV	S	4–11	2.4–6.1	5–19.7
Kohn et al.^68^	USA	NCT01852071	ADA-SCID	20	SIN LV EF1aSprom-ADA	S	-	-	24
Kohn et al.^68^	USA	NCT02999984	ADA-SCID	10	SIN LV EF1aSprom-ADA	S	-	-	24
Scaramuzza et al.^21,69^	Italy	NCT02453477	β-thalassaemia	9	GLOBE LV	M	19.5 (16.3–20)	0.7–1.5	24–48
Cavazzana-Calvo et al.^23^	France	LG001	β-thalassaemia	2	SIN LV LCR-βprom-β-globin	M	3.9	0.6	5–144
Thompson et al.^70^	USA; Australia; Thailand	NCT01745120	β-thalassaemia	18	LentiGlobin BB305 vector	M	8.1 (5.2–18.1)	0.3–1.5	34.8–61.3
Thompson et al.^70^	France	NCT02151526	β-thalassaemia	4	LentiGlobin BB305 vector	M	10.5 (8.8–13.6)	0.8–2.1	40.5–60.6
Lal et al.^71^	Multisite	NCT03207009	β-thalassaemia	11	LentiGlobin BB305 vector	M	-	1.2–4.3	2.5–20
Colvin^72^		NCT02906202	β-thalassaemia	21	LentiGlobin BB305 vector	M	-	-	0.9–26.3
Barshop^73^	USA	NCT03897361	Cystinosis	1	CTNSRD-04	M	7.88	2.07	6
AvroBio^74^	USA	FAB-201	Fabry disease	4	AVR-RD-01	M	-	-	1–1.83
AvroBio^74,75^	Canada	NCT02800070	Fabry disease	5	AVR-RD-01	M	-	-	2.67
Adair et al.^76^	USA	NCT01331018	Fanconi anemia	3	SIN LV PGKprom-FANCA	N	0.03–2.44	0.33–1.83	55.56–60
Rìo et al.^77,78^	Spain	NCT03157804	Fanconi anemia	9	SIN LV PGKprom-FANCA	N	(1.9–7.3)	0.2–0.9	2–3
Czechowicz et al.^79^	USA	NCT03814408	Fanconi anemia	2	SIN LV PGKprom-FANCA	N	0.28 (0.2–0.37)	2.08–2.21	6
Kohn et al.^80^	USA	NCT03812263	LAD	1	Chim-CD18-WPRE	M	4.2	3.8	6
Calbi et al.^81–83^	Italy	NCT01560182	MLD	29	SIN LV PGKprom-ARSA	M	10.5 (3.2–18.2)	1–7.4	3–108
Bernardo et al.^84^	Italy	NCT03488394	MPSIH	8	SIN LV PGKprom-IDUA	M	20.7 (15–29)	1–5.2	3–18
Kinsella et al.^85^	UK		MPSIIIA	1	LV.CD11b.SGSH	M	13.42	3.79	9
Walters et al.^24^	USA	NCT02140554	SCD	7	LentiGlobin BB305 vector	M	2.2 (1.6–5.1)	0.3–1.3	29.8–44.5
Walters et al.^24^	USA	NCT02140554	SCD	2	LentiGlobin BB305 vector	M	2.7 (2.2–3.2)	1.4–5	17.2–20.2
Kanter et al.^86^	USA	NCT02140554	SCD	17	LentiGlobin BB305 vector	M	4.5 (3–8)	2.8–5.6	1–20
Ribeil et al.^87^	France	NCT02151526; NCT02633943	SCD	3	LentiGlobin BB305 vector	M	4.7 (3–5.6)	0.5–1.2	25.5–52.5
Esrick et al.^88^	USA	NCT03282656	SCD	5	BCH-BB694	M	3.3–8.3	3.3–6.9	1–18
Ferrua et al.^20,89^	Italy	NCT01515462	WAS	17	LVV-w1.6 W WAS	S	12.2 (7–26.4)	0.9–4.3	6–108
Magnani et al.^90,91^	France; UK	NCT01347346; NCT01347242	WAS	9	LV-w1.6WASp	M	7.3 (2–15)	0.6–2.8	7–109
Morris et al.^92^	UK	NCT01347242	WAS	1	LV-w1.6 WASp vector	S	3.77	-	20
Labrosse et al.^93^	USA	NCT01410825	WAS	5	w1.6_hWASP_WPRE SIN-LV	S/M	9.8 (24.9–6.3)	0.53–3.3	27.6–68.4
Eichler et al.^94^	USA	NCT01896102	X-ALD	17	SIN LV MNDprom-ABCD1 (Lenti-D)	M	10.5 (6–19.4)	0.5–2.5	21.6–42
Aubourg et al.^95,96^	France		X-ALD	4	SIN LV MNDprom-ABCD1	M	-	-	60–120
Magnani et al.^97^	France	NCT02757911	X-CGD	4	G1XCGD	M	-	0.6–1.77	5–40.8
Kohn et al.^98^	UK; USA	NCT01855685; NCT02234934	X-CGD	9	SIN LV Chimericprom-CYBB	M	12.5 (6.5–32.6)	0.7–5.5	1–24
De Ravin et al.^99,100^	USA	NCT01306019	X-SCID	5	SIN LV EF1aSprom-IL-2Rγ	S	20.4 (16–25)	-	51–84
Mamcarz et al.^101,102^	USA	NCT01512888	X-SCID	11	CL20-i4-EF1α-hγc-OPT	S	8.7 (4.5–19)	0.16–1.13	1.5–33.9
Hacein-Bey-Abina et al.^103^	France; USA	NCT01410019; NCT01129544; NCT01175239	X-SCID	9	SIN gRV EF1aSprom-IL-2Rγ	N	7.8 (3.7–11.7)	0.25–2.92	12.1–38.7

*When only one value was reported it refers to the median follow-up since min-max are missing. Individual medicinal products for the same disease (i.e., encoding the same transgene) may differ for vector backbone, promoter, vector production process and transduction method.

§Oral communications at 2020 ASCGT meeting.

*PC* personal communication, *N* no conditioning, *M* myeloablative, *S* submyeloablative/non-myeloablative.

### Metanalytic results for survival

**Fig. 2:**
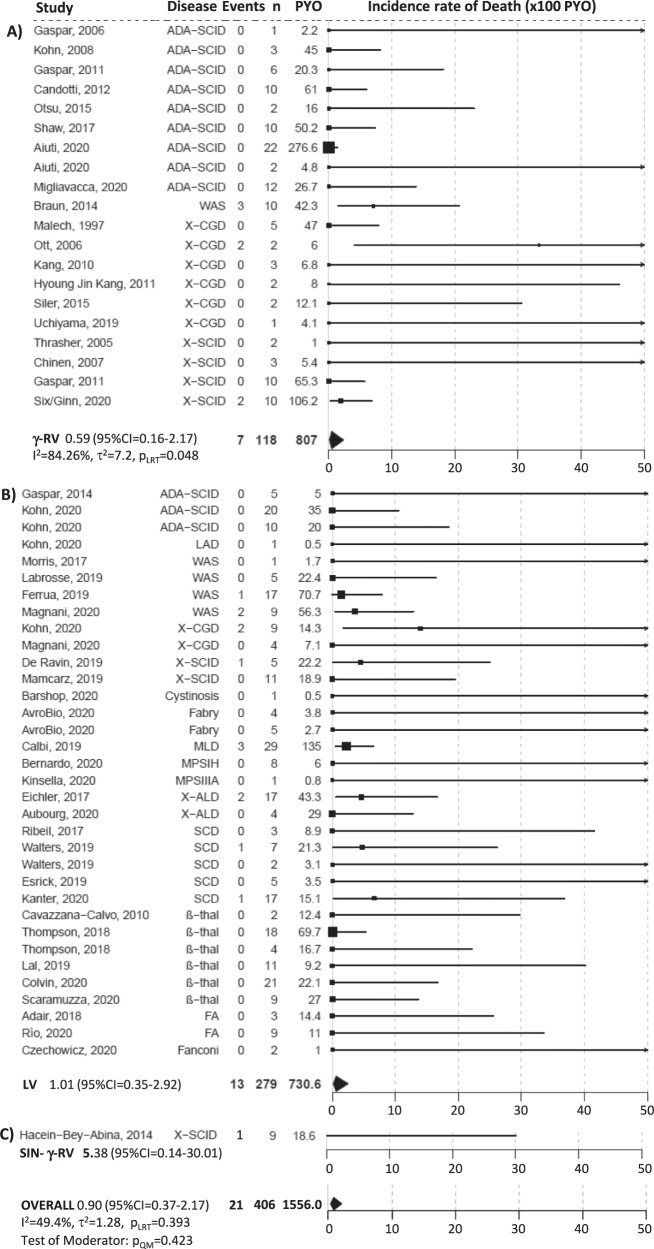
Forest plot of the incidence rate of mortality by vector type, i.e., (A) γRV, (B) LV, (C) SIN-γRV, and overall. The squares indicate the incidence rate of mortality and their size reflects the study sample size, while the horizontal lines represent 95% Confidence Intervals (CI). The diamond denotes the summary effect size from the random-effects model for all or subgroups of studies (from a meta-regression model), and the width of the diamond depicts the overall 95% CI. The indices of heterogeneity (I^2^ and τ^2^) refer to the overall analysis or to the single subgroups, and p_LRT_ is the *p* value for the test of residual heterogeneity, while p_QM_ refers to the test on vector type as moderator. All tests were two-tailed. Source data are provided as a Source Data file.

Twenty-one deaths occurred in 12 trials over a total of 1556.04 PYO for a pooled estimate of the incidence rate of death of 0.90 events per 100 PYO (95% CI = 0.37–2.17). The 21 events were observed in 13 patients treated with a LV (6 PID, 5 metabolic diseases, 2 hemoglobinopathies), 7 with a γRV [3 Wiskott–Aldrich syndrome (WAS), 2 X-linked chronic granulomatous disease(X-CGD), 2 X-linked severe combined immunodeficiency (X-SCID)] and 1 with a SIN-γRV (X-SCID). The degree of heterogeneity among studies was moderately high, although non statistically significant (I^2^ = 49.4%, τ^2^ = 1.28, *p* = 0.393). The incidence rates of death estimated in a meta-regression model were 1.01 (95% CI = 0.35–2.92) and 0.59 (95% CI = 0.16–2.17) per 100 PYO in patients treated with LV or γRV GT (*p* = 0.423) (Fig. 2). Similar results were obtained in the sensitivity analyses (Supplementary Text). The overall survival estimate at 5 years in 260 subjects with individual data (Supplementary Table 3) was 91.1% (95% CI = 86.8–95.6%) (Supplementary Fig. 1A) and similar for all vectors (*p* = 0.2652) (Supplementary Fig. 1B) and disease subgroups (PID, metabolic, haemoglobinopathies, FA; *p* = 0.7264) (Supplementary Fig. 1C). The survival profiles of the immunodeficiencies were significantly different (*p* = 0.0141) and ranged, at 5 years, from 100% for ADA-SCID to 78.8% (95% CI = 61.2–100%) for X-CGD (Supplementary Fig. 1D). The causes of death were secondary to oncogenic events in 6 cases (5 related and 1 non related to GT), infectious and bleeding complications (*n* = 8), progressions of a neurodegenerative disorder (*n* = 4), ischemic stroke (*n* = 1), cardiovascular disease (*n* = 1) and not obtainable in one case (see Supplementary Table 4 describing the patients who died). The median time to event in 19 out of the 21 deaths was 1.83 years with a range of 0.08–5 years (I–III quartiles = 0.46–3.7).

### Metanalytic results for genotoxicity

**Fig. 3:**
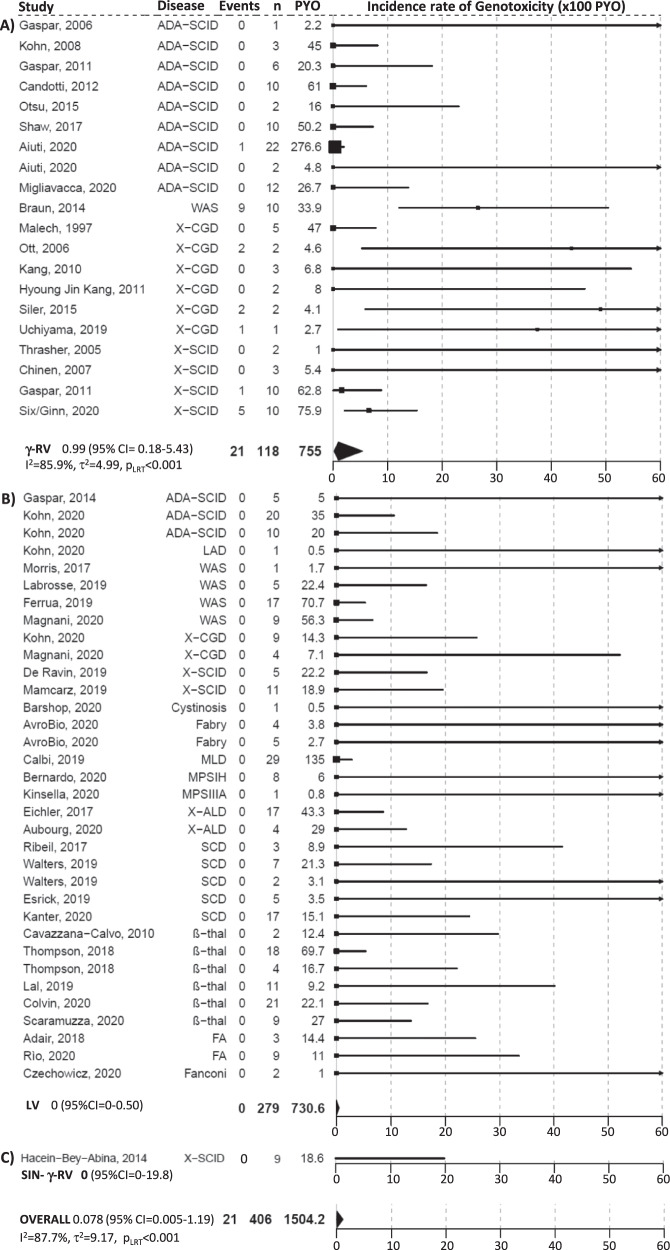
Forest plot of the incidence rate of genotoxicity by vector type, i.e., (A) γRV, (B) LV, (C) SIN-γRV, and overall. The squares indicate the incidence rate of genotoxicity and their size reflects the study sample size, while the horizontal lines represent 95% Confidence Intervals (CI). The diamond denotes the summary effect size from the random-effects model for all or subgroups of studies (from a meta-regression model), and the width of the diamond depicts the overall 95% CI. The indices of heterogeneity (I^2^ and τ^2^) refer to the overall analysis or to the single subgroups, and p_LRT_ is the p-value for the test of residual heterogeneity, while p_QM_ refers to the test on vector type as moderator. All tests were two-tailed. Source data are provided as a Source Data file.

Among the 406 patients treated, 21 genotoxic events were reported over a total of 1504.02 PYO for a pooled estimated incidence rate of 0.078 events per 100 PYO (95% CI = 0.005–1.19). All the genotoxic events occurred in 7 trials that used γRV, specifically in 9 WAS, 6 X-SCID, 5 X-CGD, and 1 ADA-SCID patients (460.6 PYO). The results of the meta-analysis indicated a very high and significant between-study heterogeneity (I^2^ = 87.7%, τ^2^ = 9.17, *p* < 0.001) that was still confirmed when restricting the analyses to γRV trials (I^2^ = 85.9%, τ^2^ = 4.99, *p* < 0.001). The pooled incidence rate obtained in this subgroup was 0.99 events per 100 PYO (95% CI = 0.18–5.43). The forest plot of the trial specific incidence rates stratified by vector type is reported in Fig. 3. The type of conditioning regimen did not result as a moderator in the meta-regression analysis (*p* = 0.440). All these results were robust to sensitivity analyses (Supplementary Text).

The characteristics of 19 out of the 21 patients experiencing a genotoxic event are reported in Supplementary Table 5. Their median age at gene-therapy was 3 years (min–max = 1 months-27 years; 3 patients were adults), while the CD34+ cell dose and VCN mean values (±sd) were 13.8 × 10^6^/kg (±7.1) and 2.4 copies/genome (±1.1), respectively. The median time to onset of genotoxic event was 2.8 years with a range of 0.7–14.8 years (I–III quartiles = 2.3–3.8). The most frequently targeted genes by oncogenesis-related γRV insertion sites were reported to be LMO2 (9 patients) and MECOM (6 patients, of whom 5 were X-CGD). Twelve patients received an allogeneic HSCT after a median of 13.9 months from the occurrence of the genotoxic event (min–max = 3.2–24.7) and 4 subsequently died (median = 18.8, min–max = 8.2–30 months from the genotoxic event), while an additional patient died without HSCT.

**Fig. 4:**
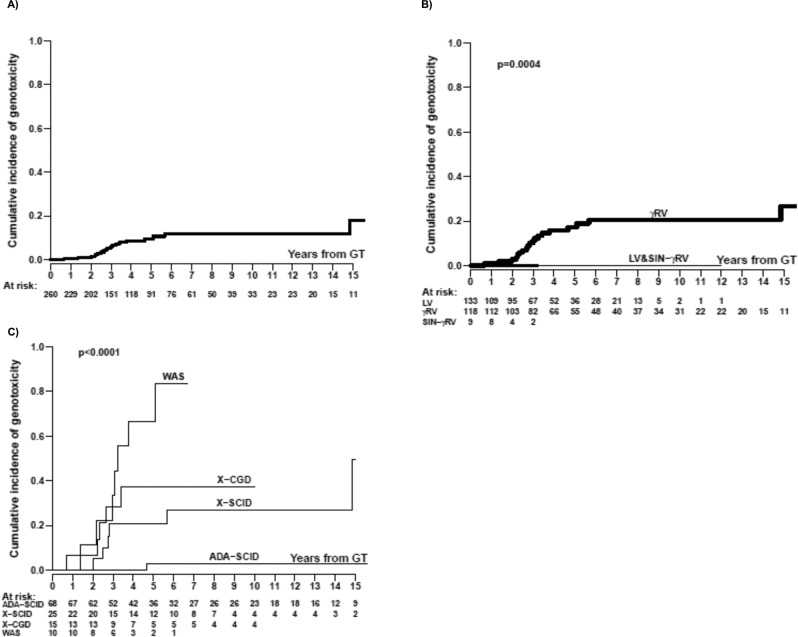
Aalen-Johansen crude cumulative incidence rate of genotoxicity (A) overall and stratified by (B) vector type and (C) disease using γRV. The result of the Gray test (two-tailed) is also reported. Source data are provided as a Source Data file.

The overall crude cumulative incidence of genotoxicity at 5 years from GT obtained from the available individual data was 9.6% (95% CI = 5.9–15.5%) (Fig. 4A). When the estimation was done stratifying by vector type we obtained 17.3% (95% CI = 11.0–27.3%) for γRV, while no event was observed in LV and SIN-γRV subgroups (*p* = 0.0004) (Fig. 4B). The curves by disease within the γRV trials show at 5 years the lowest incidence in ADA-SCID (2.7%, 95% CI = 0.3–19.2%) as compared to WAS (66.7%, 95% CI = 39.8–100.0%), X-CGD (37.2%, 95% CI = 17.6–78.4%) and X-SCID (20.6%, 95% CI = 8.3–50.7%) and this difference was maintained overtime (*p* < 0.0001) (Fig. 4C).

### Metanalytic results for engraftment

**Fig. 5:**
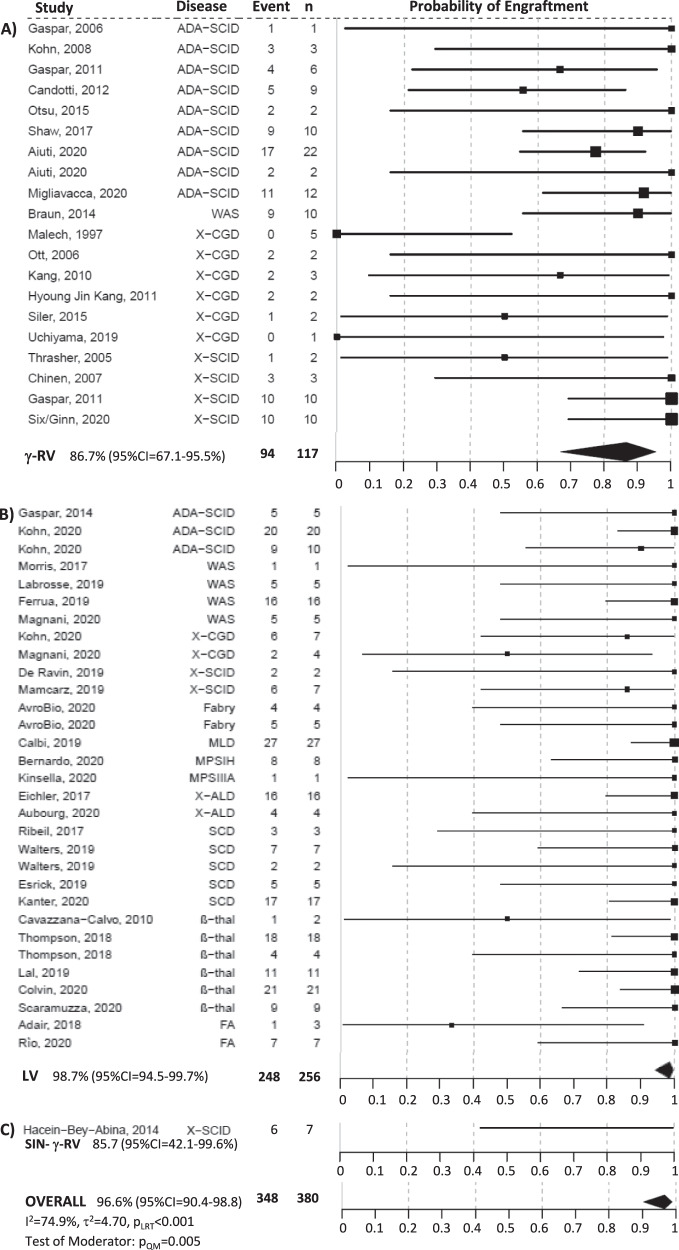
Forest plot of the rate of engraftment by vector type, i.e., (A) γRV, (B) LV, (C) SIN-γRV, and overall. The squares indicate the rate of engraftment and their size reflects the study sample size, while the horizontal lines represent 95% Confidence Intervals (CI). The diamond denotes the summary effect size from the random-effects model for all or subgroups of studies (from a meta-regression model), and the width of the diamond depicts the overall 95% CI. The indices of heterogeneity (I^2^ and τ^2^) refer to the overall analysis or to the single subgroups, and p_LRT_ is the *p* value for the test of residual heterogeneity, while p_QM_ refers to the test on vector type as moderator. All tests were two-tailed. Source data are provided as a Source Data file.

Out of the 380 patients included in the 52 trials with information available on engraftment, 348 displayed engraftment of gene corrected cells at one year, with a pooled estimate of 96.6% (95% CI = 90.4–98.8%). The rate of engrafted patients was highly heterogeneous between studies (I^2^ = 74.94%, τ^2^ = 4.70, *p* < 0.001) and the results of the regression model indicated that the viral vector was a significant moderator (*p* = 0.005), even when adjusting for conditioning (*p* = 0.020). Only in 6 trials and 8 patients treated with a LV the engraftment was lost, while this happened in 11 trials and 23 patients using a γRV. The pooled rates of engraftment were 98.7% (95% CI = 94.5–99.7%) and 86.7% (95% CI = 67.1–95.5%) for LV and γRV, respectively (I^2^ = 68.82%, τ^2^ = 3.21, *p* = 0.001) (Fig. 5). No major changes were observed in the results of the sensitivity analyses (Supplementary Text).

We next analysed myeloid cell engraftment, which is associated with HSPCs engraftment, and T cell engraftment, which reflects long-living lymphoid progenitors and mature lymphocytes and may be biased by a selective advantage in PID (Supplementary Text). Quantitative data on a limited subset of studies (and patients) was available for the specific myeloid (146 patients in 22 studies) and T cell (129 patients in 20 studies) compartments (Supplementary Table 3). Eighty out of the 146 patients with data available presented a robust myeloid engraftment (i.e., percentage of corrected cells ≥ 10% or VCN ≥ 0.1) at one year, with a pooled estimate of 47.9% (95% CI = 7.8–90.9%) and an high heterogeneity between studies (I^2^ = 93.4%, τ^2^ = 24.3, *p* < 0.001). Vector type resulted a moderator (*p* = 0.014 and *p* = 0.021 in the meta-regression model that accounted or not for conditioning, respectively), with a pooled rate of engraftment of 93.1% (95% CI = 43.0–99.6%) for LV and 4.6% (95% CI = 0.2–51.8%) for γRV (I^2^ = 89.0%, τ^2^ = 14.3, *p* < 0.001) (Supplementary Fig. 2). With regards to the lymphoid lineage, 111 patients out of the 129 with available data had a robust engraftment, with a pooled estimate of 91.9% (95% CI = 64.3–98.6%) and high heterogeneity between studies (I^2^ = 84.9%, τ^2^ = 8.8, *p* < 0.001). Results of the meta-regression model in lymphoid cells indicated that the viral vector was not a moderator (*p* = 0.284 and *p* = 0.330 in the model that accounted or not for conditioning, respectively), with pooled estimates of 95.6% (95% CI = 63.6–99.6%) and 79.0% (95% CI = 29.8–97.1%) in LV and γRV, respectively (Supplementary Fig. 3). The descriptive analyses of the individual engraftment levels in myeloid cells was consistent with a more robust engraftment associated with LV-transduced HSPC and conditioning, while these differences were less apparent within the lymphoid compartment (Supplementary Fig. 4).

## Discussion

The purpose of HSPC-GT for monogenic disorders is to achieve permanent correction of long-term repopulating cells. Here, we gathered results from 55 studies including 406 participants, using gene addition with integrating vectors, showing an extraordinary progress in the treament of genetic diseases in the past two decades. This meta-analysis provides useful information on important safety aspects of HSPC-GT across different vector platforms.

From the survival point of view, our results on early transplant-related mortality represent a favourable finding compared to allogeneic HSCT that historically has been reported in the range of 7–20% in pediatric subjects^5,6^ and 6–14% in adolescent and adults^7,8^ due to toxicity, infections and acute GvHD. Of the 21 deaths reported, apart from those caused by genotoxicities, which were all derived from γRV trials, the others were mainly due to concomitant infections, progression of neurodegenerative disease or acute events not related to GT. The type of vector does not seem to be a moderator in the meta-analysis, since the three different vectors have a similar behavior in terms of survival. The overall survival at 5 years post GT was 91.1% without relevant differences among disease subgroups. In allogeneic HSCT, which currently represents the best available option of a definitive treatment for most severe disorders, the 5-year survival has been reported to be 74% for PID^9^, 73% for FA^10^, 59–95% for metabolic diseases^11–13^ such as MLD and MPSI, respectively, and 91–92% for haemoglobinopathies^14,15^. Comparison between GT and allogeneic HSCT, however, was not the objective of this work and will require additional data collection and specific analyses. Registries of the European Society for Blood and Marrow Transplantation (EBMT) or the Center for International Blood and Marrow Transplant Research (CIBMTR) could represent a potential platform for comparing allogeneic HSCT and GT but currently are not designed to retrieve sufficiently high quality data for long-term monitoring and GT-related parameters.

Oncogenic events related to the insertional mutagenesis occurred in 21 patients over a total of 1504.02 PYO for a pooled overall incidence rate of 0.078 events per 100 PYO. Remarkably, 84% of oncogenic events occurred within the first five years post-GT, regardless of the type of disease, but the occurrence of one case 15 years after GT suggests that long-term follow-up should be implemented at least until this time point, in line with current EMA guidelines^16^. Post-marketing pharmacovigilance should be able to eventually capture signals deriving from HSPC-GT at longer time, even life-long.

The oncogenic events appear to be the results of a multistep process, in which the initial hit, in most cases an integration from a γRV vector near the *LMO2* gene activating its constitutive transcription, is followed by rearrangements, chromosomal translocations and other somatic mutations. Incidence of genotoxicity in γRV studies ranged from 0.20 events per 100 PYO in ADA-SCID patients to 26.6 events per 100 PYO in WAS patients. The different incidence among trials and diseases suggests that there are additional factors, including transgene function, disease background, vector dose, and individual genetic predisposition that influence the likelihood of occurrence of transformation. The molecular defect that causes inborn errors of immunity per se may predispose to tumorigenesis with variable degree, depending on the underlying molecular mechanisms^17^, together with an impaired tumor immune surveillance^18^.

Unlike γRVs which contain strong retroviral enhancer and promoter elements (within the proviral long-terminal repeats; LTR) capable of transactivating of neighboring genes, LVs are designed with self-inactivating transcriptionally silent LTRs and often carry relatively weak or lineage-specific internal cellular promoters. These genetic features, together with different insertion site preferences from γRV, may provide a mechanistic explanation for the lack of reported malignant clonal expansion in LV trials. This observation substantiates with a robust clinical follow-up (730.7 PYO) the superior LV biosafety profile predicted by multiple non-clinical studies including in tumor prone mice^19^ and well correlates with the lack of clonal perturbation assessed by insertion site analyses in LV-based trials^20–22^. One patient in a LV trial for ß-thalassemia was reported to show a dominant clone harbouring an integration in the *HMGA2* gene, causing deregulation of HMGA2 expression which, however, was not associated with adverse effects^23^. Following the data cut off of our analyses, two cases of AML were reported in patients treated in a phase 1/2 (HGB-206) study with bb1111 LV GT for sickle cell disease (SCD)^24,25^. The review from regulatory authorities found that the viral vector was unlikely to be the cause since in one of the patients the vector was not detectable in the leukemic cells while in the other it was found in a genomic site (*VAMP4*) which does not appear to be involved in tumor development^26^. Very recently, a patient treated with elivaldogene autotemcel (Lenti-D) in the ALD-104 study for X-linked adrenoleukodystrophy was diagnosed with myelodysplastic syndrome, likely mediated by LV insertion^27^. Emerging technology platforms based on targeted gene editing should in principle further reduce the residual potential low risk of insertional mutagenesis associated with genome-wide integration of LVs^22,28,29^. However, larger studies and longer follow-up are needed to carefully assess the clinical efficacy and safety of gene editing based approaches. The occurrence of a secondary tumor (myelodysplasia followed by leukemia) in one SCD patient treated with LV^30^, likely as a result of chemotherapy-induced mutagenesis on residual host cells as well as a bone marrow dysplasia observed in an ADA-SCID patient treated with γRV deriving from non-corrected cells^31^ were not unexpected. Indeed, the risk of secondary tumors is reported to be 4% at 7 years after autologous HSCT, with a median onset of 2.5 years post-transplantation (range = 3 months-7 years). The risk may be higher in immunodeficient patients or in conditions characterized by hematopoietic stress and history of previous treatment with cytotoxic drugs, such as in SCD^32^. In this regard, replacement of standard chemotherapy with non genotoxic conditioning based on depleting antibodies or immunotoxins could reduce this risk^33,34^.

In the majority of patients, gene modified cells persisted long-term (≥one year), indicating the ability of infused HSPCs to engraft, self-renew and differentiate. We found that the nature of the vector represents a moderator of this parameter, also adjusted for conditioning, confirming, so far in the clinical setting, the higher efficiency of LV in transducing repopulating hematopoietic stem cells. On the other hand, the selective advantage of functionally corrected cells in PID subjects may compensate for the the lower transduction when adopting the γRV platform. Conversely, conditioning regimen alone had no role as moderator (*p* = 0.149). However, it should be considered that the infusion of corrected HSPCs in absence of conditioning was mainly chosen for diseases in which a selective advantage for gene corrected lymphoid cells (SCID) or HSPCs (FA) was expected thanks to the selective advantage at the level of progenitor and/or mature cells, and this could alleviate the need for a chemotherapy regimen.

The creation of a dedicated global registry will be instrumental to allow comprehensive analyses of the outcome of HSPC-GT across different diseases. At present, there is still debate on the optimal format of registries that could monitor long-term safety and efficacy of ATMPs, in compliance to requests of regulatory authorities and payors. These registries could collect data on specific ATMPs or diseases but their accessibility could still be limited and there are known difficulties in harmonization between countries. Existing infrastructure such as the one used by EBMT could retrieve data on all HSPC-GT procedures and allow comparison with HSCT. This approach has been used to capture information on long-term follow-up of patients treated with CAR-T cells, but its success and broader applicability are still under evaluation^35^.

We acknowledge that our study has some potential limitations due to the evolving nature of GT, for example the lack of conditioning in early studies with γRV for PID, and due to the focus on several small trials but this is a specificity of a therapeutic approach that has been almost entirely devoted until now to rare diseases and/or is still in its early phase of clinical development. We also took in consideration the fact that the follow-up is not homogeneously updated, and therefore we conducted a sensitivity analysis on studies with an adequate follow-up that confirmed our results. While we are confident that at the time of data cut all genotoxic events up to date have been reported, some deaths might have been missed if not properly reported. We also recognize that engraftment is not a hard clinical endpoint for efficacy, but traditional efficacy endpoints are disease specific and this would have precluded the meta-analytic approach that combines all diseases. The creation of a dedicated global registry will be instrumental to allow comprehensive prospective meta-analyses of the outcome of HSPC-GT across different diseases. In conclusions, results from this meta-analysis summarizing two decades of studies on HSPC-GT in over 400 patients shows stable reconstitution of haematopoiesis with gene-corrected cells in most recipients and superior engraftment and safer genotoxic profile in patients receiving LV-transduced HSPCs.

## Methods

### Search strategy and selection criteria

In this systematic review and meta-analysis, we followed PRISMA guidelines. Searches were conducted in PubMed, Embase and Cochrane Central Register of Controlled Trials to identify potentially eligible literature from inception to October 2020. The search strategy used the following search terms in combination: “genetic disease” and “GT” or “ex-vivo GT”, “autologous hematopoietic stem cell transplantation” or “HSPC-GT” (Supplementary Text). We also handsearched the reference lists of every selected study and assessed relevant studies for further publications. A search on ClinicalTrials.gov was performed to identify potential missing trials from the original evaluations. Corresponding authors of selected publications were contacted to ask clarification and retrieve missing data. In addition, reviews, conference abstracts and oral communications were identified by electronic searching and included as “gray literature data”. Abstracts of articles were then independently reviewed by two authors (AA and FT) and the full text was obtained for suitable articles. Data were also extracted independently and stored in a Excel file (Excel 2016).

To be eligible, studies must have: (1) included patients affected by monogenic inherited diseases treated with HSPC-GT; (2) reported outcomes, including numbers of deaths, genotoxicities and engraftments. Genotoxic events were intended as the first occurred haematological malignancy related or probably related to GT. Second malignancies and tumors not related to GT were not included in the genotoxicity analysis. Engraftment was considered successful when molecular tests reported the presence of gene corrected cells for ≥1 year post-GT by PCR for transduced cells or transgene protein expression. When available, we collected quantitative data on engrafment of the corrected cells at one year after GT both in the myeloid and T cell compartments and considered a robust engraftment when VCN was >0.1 or the percentage of corrected cells was >10% (Supplementary Text). Non clinical research and clinical studies on cancer or gene editing were excluded. Studies were also excluded if they were limited to qualitative description. In addition to the target reported outcomes, the following variables were extracted: CT registration number, disease, type of vector, type of conditioning regimen, summary measures on infused CD34+ cells/kg, vector copy number (VCN/genome) on the drug product, duration of follow-up after GT and year at the latest update. When possible, individual data on age at treatment, infused CD34+ cells/kg, VCN/genome, occurred events, timing of any subsequent HSCT and duration of follow-up post-GT were also retrieved (see Supplementary Text for more details). Individual medicinal products for the same disease (i.e., encoding the same transgene) may differ for vector backbone, promoter, vector production process and transduction method. The quality of the included studies was evaluated based on a six-item tool that assessed the selection and outcome domains (Supplementary Table 6) and was adapted from the The Newcastle-Ottawa Scale (NOS) for assessing the quality of non-randomised studies in meta-analysis^36^. A global score ranging 0–15 (from lowest to highest quality) was also obtained from the six items. All studies meeting inclusion-exclusion criteria were independently evaluated by 2 trained authors (AA and FT).

### Statistical analysis

The meta-analysis on the incidence rate of mortality and genotoxicity was conducted using a random intercept Poisson model, while the analysis on the rate of engraftment was performed by means of a random intercept logistic model^37^. The trial specific total exposures in terms of PYO were obtained from individual data or, when not available, from minimum, median and maximum follow-up. Heterogeneity across studies was graphically explored drawing forest plots and quantified by the I^2^ and τ^2^ indices, while p-values based on the likelihood ratio test were provided to test for residual heterogeneity (H_0_: τ^2^ = 0, α = 0.05, two sided). Meta-regression models were used to assess the influence of one or more moderators on the outcomes and the Cochran Q statistic was used as (omnibus) test on moderators. Summary results were reported along with their 95% Confidence Intervals (CI). A sensitivity analysis was done by excluding studies with a median follow-up less that 2 years, considered as not fully adequate.

The available individual data were described in terms of survival by means of the Kaplan–Meyer estimator and comparisons were done by the log-rank test, while the Aalen–Johansen cumulative incidence curves were used to describe genotoxicity (with death as competing event) and the Gray test was used for comparisons. Estimates were reported with the corresponding 95% CI. Analyses were performed using the software R version 3.6 (package metafor, version 2.4, for the meta-analysis).

### Reporting summary

Further information on research design is available in the Nature Research Reporting Summary linked to this article.

## Supplementary information

Supplementary Information

Reporting Summary

## Data Availability

Source data are provided with this paper. Data were extracted from previously published research listed in Table 1, and they are also available in the public domain.

## Code availability

The code to reproduce analyses is provided in https://figshare.com/articles/software/R_code_MA_in_GT_R/17712098.
