# Supplementary material for: A systematic review and meta-analysis of gene therapy with hematopoietic stem and progenitor cells for monogenic disorders
Source: Nat Commun. 2022 Mar 14;13:1315. doi: 10.1038/s41467-022-28762-2 (PMC8921234; doi:10.1038/s41467-022-28762-2)
Supplement: Supplementary file 1 — Supplementary Information [file 41467_2022_28762_MOESM1_ESM.pdf]

Supplementary material

Methods

Search Strategy (Box 1)

No language limit was applied. If multiple papers were referring to the same trial, we selected the one with the largest and more updated.

**PubMed Advanced Search Builder**	*(("genetic diseases, inborn"[MeSH Terms] OR ("genetic"[All Fields] AND "diseases"[All Fields] AND "inborn"[All Fields]) OR "inborn genetic diseases"[All Fields] OR ("genetic"[All Fields] AND "disease"[All Fields]) OR "genetic disease"[All Fields] OR (("monogenic"[All Fields] OR "monogenically"[All Fields] OR "monogenics"[All Fields]) AND ("disease"[MeSH Terms] OR "disease"[All Fields] OR "diseases"[All Fields] OR "disease s"[All Fields] OR "diseased"[All Fields]))) AND ("genetic therapy"[MeSH Terms] OR ("genetic"[All Fields] AND "therapy"[All Fields]) OR "genetic therapy"[All Fields] OR ("gene"[All Fields] AND "therapy"[All Fields]) OR "gene therapy"[All Fields])) OR ("ex"[All Fields] AND "vivo"[All Fields] AND ("genetic therapy"[MeSH Terms] OR ("genetic"[All Fields] AND "therapy"[All Fields]) OR "genetic therapy"[All Fields] OR ("gene"[All Fields] AND "therapy"[All Fields]) OR "gene therapy"[All Fields])) OR (("autolog"[All Fields] OR "autologeous"[All Fields] OR "autologic"[All Fields] OR "autological"[All Fields] OR "autologous"[All Fields] OR "autologously"[All Fields]) AND ("haematopoietic stem cell transplantation"[All Fields] OR "hematopoietic stem cell transplantation"[MeSH Terms] OR ("hematopoietic"[All Fields] AND "stem"[All Fields] AND "cell"[All Fields] AND "transplantation"[All Fields]) OR "hematopoietic stem cell transplantation"[All Fields])) OR (("haematopoietically"[All Fields] OR "hematopoietic system"[MeSH Terms] OR ("hematopoietic"[All Fields] AND "system"[All Fields]) OR "hematopoietic system"[All Fields] OR "haematopoietic"[All Fields] OR "hematopoietic"[All Fields] OR "hematopoietically"[All Fields]) AND ("plant stems"[MeSH Terms] OR ("plant"[All Fields] AND "stems"[All Fields]) OR "plant stems"[All Fields] OR "stem"[All Fields] OR "microscopy, electron, scanning transmission"[MeSH Terms] OR ("microscopy"[All Fields] AND "electron"[All Fields] AND "scanning"[All Fields] AND "transmission"[All Fields]) OR "scanning transmission electron microscopy"[All Fields]) AND ("stem cells"[MeSH Terms] OR ("stem"[All Fields] AND "cells"[All Fields]) OR "stem cells"[All Fields] OR ("progenitor"[All Fields] AND "cell"[All Fields]) OR "progenitor cell"[All Fields]) AND ("genetic therapy"[MeSH Terms] OR ("genetic"[All Fields] AND "therapy"[All Fields]) OR "genetic therapy"[All Fields] OR ("gene"[All Fields] AND "therapy"[All Fields]) OR "gene therapy"[All Fields]))*

Data Extraction

Due to the heterogeneity in the underlying disease, endpoint measures, and time of analyses, it was not possible to perform an analysis on efficacy endpoints. We therefore identified the persistence of gene corrected cells as a common variable to compare the performance of different vectors in the various diseases, regardless of the therapeutic threshold required for efficacy. The persistence of the engrafted cells was defined by qualitative evaluation as presence of gene corrected cells at 1 year post GT, as identified by PCR or protein-based assay. To perform an in-deep quantitative analysis, the engrafted cells were analyzed when PCR-based assays (VCN/genome or percentage of the corrected cells) were available. We stratified the data in the following 5 categories (Box 2) and considered as robust engraftment the 3 and 4 classes. We extrapolated these data from whole blood population, peripheral blood mononuclear cells, myeloid/progenitor cell lineage and T cell lineage.

Box 2: Legend for quantification of the engrafted corrected cells at 1 year post GT.

Class	VCN/genome	% of corrected cells
**0**	Absent/undetectable	Absent/undetectable
**1**	<0.01	<1%
**2**	0.01-0.1	1-10%
**3**	0.1-1	10-100%
**4**	>1	

In case of infusion of >1 bag of CD34+ cells with different VCN, a sum of the number of infused CD34+ and the weighted average of the VCN was calculated.

Overall collection of serious adverse event (SAE) was not the purpose of the study and deemed not feasible due the lack of detailed reporting in most of the source data used for this analyses.

Results

Methodological quality of the included studies

In evaluating the representativeness of the exposed cohort of patients to GT, only 2 (3.6%) and 4 (7.3%) studies were indicated by the two raters as involving selected groups. They represent studies where a single patient was treated, as in pilot studies (Gaspar, 2006; Kinsella, 2020; Kohn, 2020; Ribeil, 2017), or where there is a discrepancy between the cohort and the patients population size (Esrick, 2019; Ribeil, 2017). A strong agreement was observed (90.9%) between the two rates on this item, while no discrepancies were seen on the ascertainment of exposure, done on secure record in all trials, and on the outcome, which was not present at the start of all trials. There were no concerns about outcome assessment, as in all studies outcome confirmation was obtained by reference to secure records. The duration of follow-up was evaluated long enough for the outcomes to occur in 41 studies (74.6%, for a total of 323 patients) by the two raters, while in 14 trials (25.4%, for a total of 83 patients) the median follow-up was not fully adequate (i.e. <2 years) since they were the most recently initiated (Thrasher 2005; Morris 2017; Lal 2019; Colvin 2020; Kanter 2020; Barshop 2020; AvroBio 2020; Magnani XCGD 2020; Czechowicz 2020; Kinsella 2020, Bernardo 2020; Gaspar 2014; Esrick 2019, Kohn LAD 2020). Finally, the two raters agreed on considering 4 studies (7.3%, for a total of 15 patients) with no statement about the follow-up, even if we recovered information on exposure to genotoxicity, death and on loss of engraftment. (Adair, 2017, Czechowicz 2020, https://avrobiofabrytrial.com/). Only one study (Hacein-Bey-Abina 2010) (1.8%, 10 patients) presented <20% of patients who were lost to follow-up, but it contributed with a relevant piece of exposure (i.e. 106.2 PYO). The median total score was 14 (min-max=10-15) for both the raters. Due to the presence of trials with a short follow-up, we performed the sensitivity analyses.

Sensitivity analyses

Survival

In the sensitivity analysis including only the 41 trials with an adequate follow-up (i.e. median follow-up <2 years), the overall pooled incidence rate of death was 0.87 events per 100 PYO (95%CI=0.33-2.23) (I^2=55.1%, τ^2=1.46, p=0.083). The results of the meta-regression model including vector type are: 1.01 deaths per PYO (95%CI=0.32-3.16) and 0.54 (95%CI=0.13-2.22) for LV and γRV, respectively (p=0.396) (I^2=56.7%, τ^2=1.60).

Genotoxicity

The estimated pooled overall incidence rate was 0.10 genotoxic events per 100 PYO (95% CI= 0.008-1.35) (I^2=88.6%, τ^2=8.97, p <0.001) and 1.00 events per 100 PYO (95%CI=0.18-5.51) in the subgroup of γRV trials (I^2=86.4%, τ^2=5.06, p <0.001).

Engraftment

The pooled overall rate of engraftment was 95.4% (95%CI=87.7-98.4%) (I^2=72.61%, τ^2=3.95, p<0.001). Engraftment was successfully retained with a rate of 98.3% (95%CI=92.7-99.6%) and 87.2%, (95%CI=68.5-95.5%) in patients treated with an LV and γRV, respectively (p=0.014 and p=0.038 in the model that accounted or not for conditioning, respectively).

Quantitative analysis of the engrafted cells

The distribution of the engrafted cells evaluated in ordered categories was performed in the whole blood population and peripheral blood mononuclear cells (data not shown) and in the myeloid/progenitor cell and T cell lineages and was also stratified by conditioning. In the myeloid compartment (Supplementary Figure 4A), overall we retrieved 146 patients of whom 64 (43.8%) and 82 (56.1%) treated with γRV and LV, respectively while in the lymphoid compartment (Supplementary Figure 4B), 129 patients of whom 46 (37.2%) were treated with γRV and 75 (58.1%) with LV vector.

Supplementary Figure 1: Curves of survival obtained from the individual available data A) overall and stratified by B) vector type, C) disease subgroups and D) type of PID

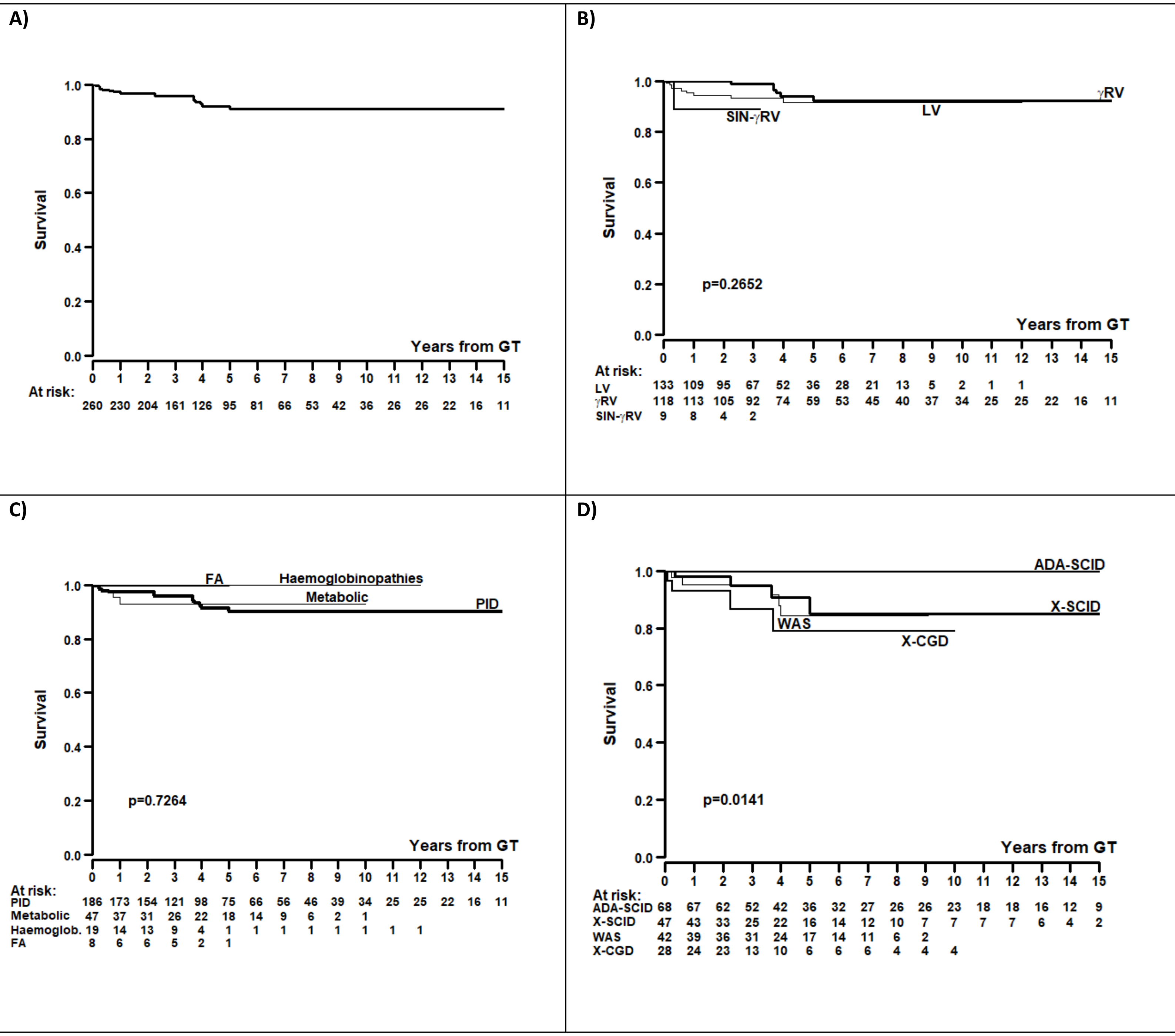

Supplementary Figure 2: Forest plot of the rate of robust engraftment in the myeloid compartment by vector type, i.e. (A) γRV, (B) LV, and overall. The squares indicate the rate of engraftment and their size reflects the study sample size, while the horizontal lines represent 95% Confidence Intervals (CI). *The diamond denotes the summary effect size for the random-effects model for all or subgroup of studies (from a meta-regression model), and the width of the diamond depicts the overall 95% CI. The indices of heterogeneity* (I^2 and τ^2) *refer to the overall analysis or to the single subgroups, and* p_{LRT} *is the p-value for the test of residual heterogeneity, while* p_{QM} *to the test on vector type as moderator.* All tests were two-tailed. Source data are provided as a Source Data file.

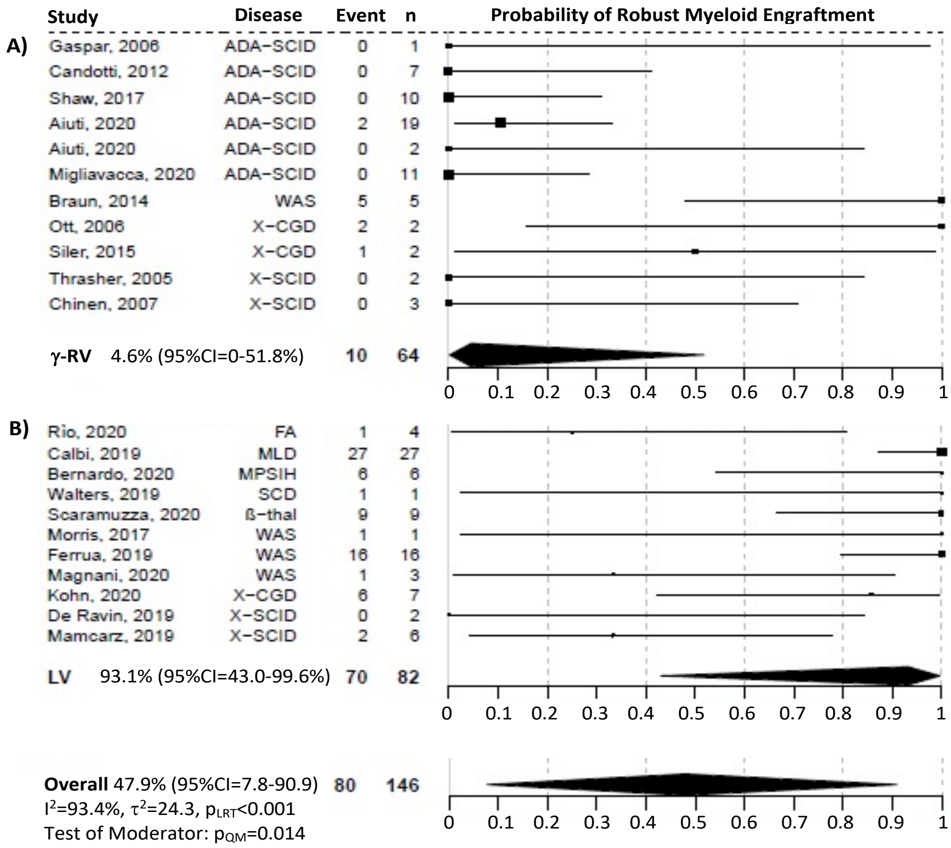

Supplementary Figure 3: Forest plot of the rate of robust engraftment in the lymphoid compartment by vector type, i.e. (A) γRV, (B) LV, and overall. The squares indicate the rate of engraftment and their size reflects the study sample size, while the horizontal lines represent 95% Confidence Intervals (CI). *The diamond denotes the summary effect size for the random-effects model for all or subgroup of studies (from a meta-regression model), and the width of the diamond depicts the overall 95% CI. The indices of heterogeneity* (I^2 and τ^2) *refer to the overall analysis or to the single subgroups, and* p_{LRT} *is the p-value for the test of residual heterogeneity, while* p_{QM} *to the test on vector type as moderator.* All tests were two-tailed. Source data are provided as a Source Data file.

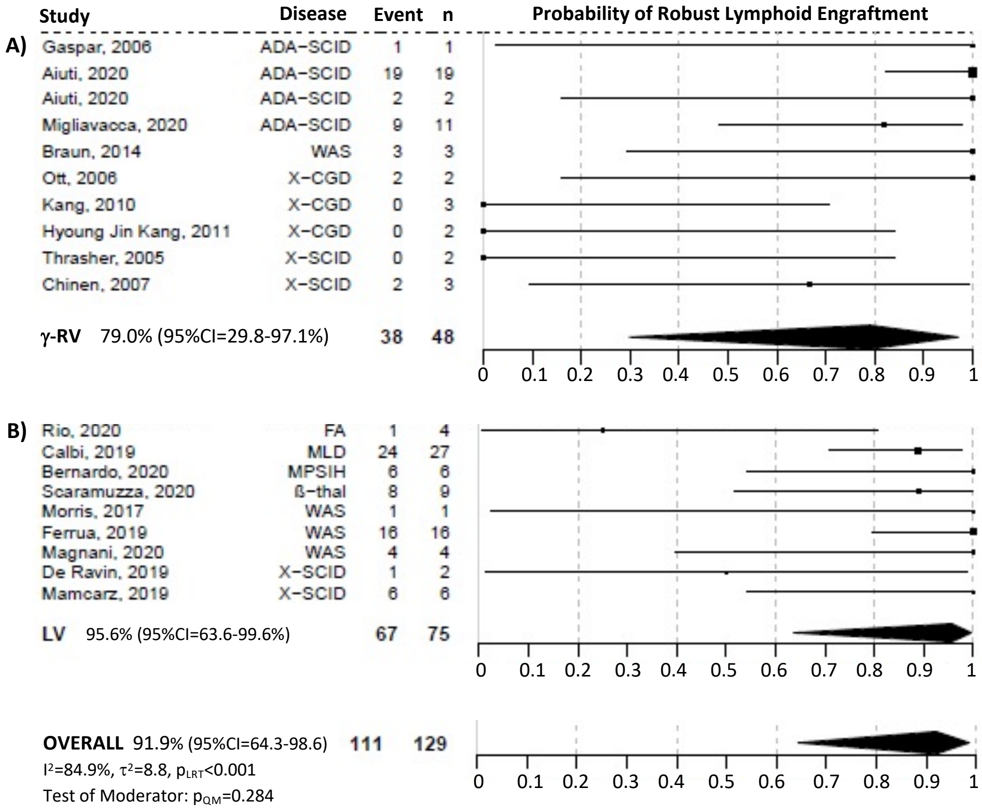

Supplementary Figure 4: Distribution of the engrafted levels in the A) myeloid/progenitor cell and in the B) T cell lineages among the 3 different vector types, overall and stratified by type of conditioning

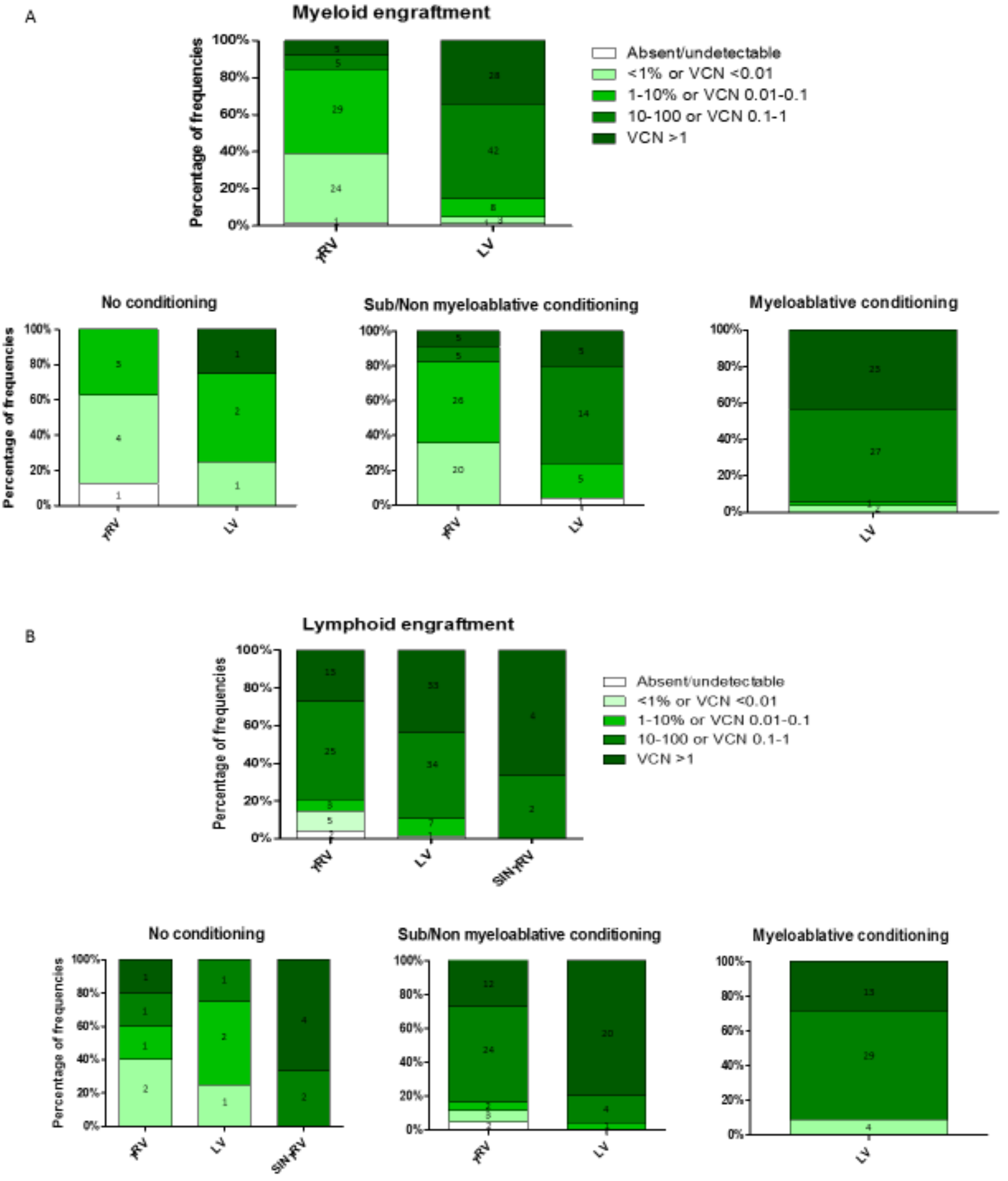

Supplementary Table 1: Data on the methodological quality of the included studies assessed by 2 authors (A and B)

A) 1st author (FT)

Publication	Disease	Selection 1	Selection 2	Selection 3	Outcome 1	Outcome 2	Outcome 3	Total
Aiuti, 2020	ADA-SCID	3	3	1	3	1	4	15
Aiuti, 2020	ADA-SCID	2	3	1	3	1	4	14
Candotti, 2012	ADA-SCID	3	3	1	3	1	4	15
Gaspar, 2006	ADA-SCID	1	3	1	3	1	4	13
Gaspar, 2011	ADA-SCID	3	3	1	3	1	4	15
Gaspar, 2014	ADA-SCID	2	3	1	3	0	4	13
Migliavacca, 2020	ADA-SCID	2	3	1	3	1	4	14
Shaw, 2017	ADA-SCID	3	3	1	3	1	4	15
Kohn, 2008	ADA-SCID	3	3	1	3	1	4	15
Kohn, 2020	ADA-SCID	3	3	1	3	1	4	15
Kohn, 2020	ADA-SCID	3	3	1	3	1	4	15
Otsu, 2015	ADA-SCID	2	3	1	3	1	4	14
Cavazzana-Calvo, 2010	β-thalassaemia	2	3	1	3	1	4	14
Colvin, 2020	β-thalassaemia	3	3	1	3	0	4	14
Lal, 2019	β-thalassaemia	3	3	1	3	0	4	14
Scaramuzza, 2020	β-thalassaemia	3	3	1	3	1	4	15
Thompson, 2018	β-thalassaemia	3	3	1	3	1	4	15
Thompson, 2018	β-thalassaemia	3	3	1	3	1	4	15
Barshop, 2020	Cystinosis	2	3	1	3	0	4	13
AvroBio, 2020	Fabry disease	2	3	1	3	0	1	10
AvroBio, 2021	Fabry disease	2	3	1	3	1	1	11
Adair, 2018	FA	3	3	1	3	1	1	12
Czechowicz, 2020	FA	3	3	1	3	0	1	11
Rìo, 2020	FA	3	3	1	3	1	4	15
Kohn, 2020	LAD	1	3	1	3	0	4	12
Calbi, 2019	MLD	3	3	1	3	1	4	15
Bernardo, 2020	MPSIH	3	3	1	3	0	4	14
Kinsella, 2020	MPSIIIA	1	3	1	3	0	4	12
Esrick, 2019	SCD	1	3	1	3	0	4	12
Kanter, 2020	SCD	3	3	1	3	0	4	14
Ribeil, 2017	SCD	2	3	1	3	1	4	14
Walters, 2019	SCD	3	3	1	3	1	4	15
Walters, 2019	SCD	3	3	1	3	1	4	15
Ferrua, 2019	WAS	3	3	1	3	1	4	15
Labrosse, 2019	WAS	3	3	1	3	1	4	15
Magnani, 2020	WAS	3	3	1	3	1	4	15
Morris, 2017	WAS	2	3	1	3	0	4	13
Braun, 2014	WAS	3	3	1	3	1	4	15
Aubourg, 2020	X-ALD	3	3	1	3	1	4	15
Eichler, 2017	X-ALD	3	3	1	3	1	4	15
Kang, 2010	X-CGD	2	3	1	3	1	4	14
Kohn, 2020	X-CGD	3	3	1	3	1	4	15
Magnani, 2020	X-CGD	3	3	1	3	0	4	14
Malech, 1997	X-CGD	3	3	1	3	1	4	15
Siler, 2015	X-CGD	2	3	1	3	1	4	14
Uchiyama, 2019	X-CGD	2	3	1	3	1	4	14
Kang, 2011	X-CGD	2	3	1	3	1	4	14
Ott, 2006	X-CGD	2	3	1	3	1	4	14
Chinen, 2007	X-SCID	2	3	1	3	1	4	14
De Ravin, 2019	X-SCID	2	3	1	3	1	4	14
Gaspar, 2011	X-SCID	3	3	1	3	1	4	15
Hacein-Bey-Abina, 2014	X-SCID	3	3	1	3	1	4	15
Mamcarz, 2019	X-SCID	3	3	1	3	1	4	15
Six, 2020	X-SCID	3	3	1	3	1	3	14
Thrasher, 2005	X-SCID	2	3	1	3	0	4	13

B) 2nd author (AA)

Publication	Disease	Selection 1	Selection 2	Selection 3	Outcome 1	Outcome 2	Outcome 3	Total
Aiuti, 2020	ADA-SCID	3	3	1	3	1	4	15
Aiuti, 2020	ADA-SCID	2	3	1	3	1	4	14
Candotti, 2012	ADA-SCID	3	3	1	3	1	4	15
Gaspar, 2006	ADA-SCID	2	3	1	3	1	4	14
Gaspar, 2011	ADA-SCID	3	3	1	3	1	4	15
Gaspar, 2014	ADA-SCID	3	3	1	3	0	4	14
Migliavacca, 2020	ADA-SCID	2	3	1	3	1	4	14
Shaw, 2017	ADA-SCID	3	3	1	3	1	4	15
Kohn, 2008	ADA-SCID	3	3	1	3	1	4	15
Kohn, 2020	ADA-SCID	3	3	1	3	1	4	15
Kohn, 2020	ADA-SCID	3	3	1	3	1	4	15
Otsu, 2015	ADA-SCID	2	3	1	3	1	4	14
Cavazzana-Calvo, 2010	β-thalassaemia	2	3	1	3	1	4	14
Colvin, 2020	β-thalassaemia	3	3	1	3	0	4	14
Lal, 2019	β-thalassaemia	3	3	1	3	0	4	14
Scaramuzza, 2020	β-thalassaemia	3	3	1	3	1	4	15
Thompson, 2018	β-thalassaemia	3	3	1	3	1	4	15
Thompson, 2018	β-thalassaemia	3	3	1	3	1	4	15
Barshop, 2020	Cystinosis	2	3	1	3	0	4	13
AvroBio, 2020	Fabry disease	2	3	1	3	0	1	10
AvroBio, 2021	Fabry disease	2	3	1	3	1	1	11
Adair, 2018	FA	3	3	1	3	1	1	12
Czechowicz, 2020	FA	3	3	1	3	0	1	11
Rio, 2020	FA	3	3	1	3	1	4	15
Kohn, 2020	LAD	1	3	1	3	0	4	12
Calbi, 2019	MLD	3	3	1	3	1	4	15
Bernardo, 2020	MPSIH	3	3	1	3	0	4	14
Kinsella, 2020	MPSIIIA	2	3	1	3	0	4	13
Esrick, 2019	SCD	2	3	1	3	0	4	13
Kanter, 2020	SCD	3	3	1	3	0	4	14
Ribeil, 2017	SCD	1	3	1	3	1	4	13
Walters, 2019	SCD	3	3	1	3	1	4	15
Walters, 2019	SCD	3	3	1	3	1	4	15
Ferrua, 2019	WAS	3	3	1	3	1	4	15
Labrosse, 2019	WAS	3	3	1	3	1	4	15
Magnani, 2020	WAS	3	3	1	3	1	4	15
Morris, 2017	WAS	2	3	1	3	0	4	13
Braun, 2014	WAS	3	3	1	3	1	4	15
Aubourg, 2020	X-ALD	3	3	1	3	1	4	15
Eichler, 2017	X-ALD	3	3	1	3	1	4	15
Kang, 2010	X-CGD	2	3	1	3	1	4	14
Kohn, 2020	X-CGD	3	3	1	3	1	4	15
Magnani, 2020	X-CGD	3	3	1	3	0	4	14
Malech, 1997	X-CGD	3	3	1	3	1	4	15
Siler, 2015	X-CGD	2	3	1	3	1	4	14
Uchiyama, 2019	X-CGD	2	3	1	3	1	4	14
Kang, 2011	X-CGD	2	3	1	3	1	4	14
Ott, 2006	X-CGD	2	3	1	3	1	4	14
Chinen, 2007	X-SCID	2	3	1	3	1	4	14
De Ravin, 2019	X-SCID	2	3	1	3	1	4	14
Gaspar, 2011	X-SCID	3	3	1	3	1	4	15
Hacein-Bey-Abina, 2014	X-SCID	3	3	1	3	1	4	15
Mamcarz, 2019	X-SCID	3	3	1	3	1	4	15
Six, 2020	X-SCID	3	3	1	3	1	3	14
Thrasher, 2005	X-SCID	2	3	1	3	0	4	13

Supplementary Table 2: Distribution of the number of studies and patients by disease and vector type

Disease groups	Disease	LV #studies	LV # pts	γRV #studies	γRV # pts	SIN-γRV #studies	SIN-γRV # pts	TOTAL #studies	TOTAL # pts
**PID**	**ADA-SCID**	3	35	9	68			12	103
	**LAD**	1	1					1	1
	**WAS**	4	32	1	10			5	42
	**X-CGD**	2	13	6	15			8	28
	**X-SCID**	2	16	4	25	1	9	7	50
	**Total**	12	97	20	118	1	9	33	224
**IBMFS**	**Fanconi**	3	14					3	14
**Hemaglobinopathies**	**ß-thalassemia**	6	65					6	65
	**Sickle cell**	5	34					5	34
	**Total**	11	99					11	99
**Metabolic diseases**	**Cystinosis**	1	1					1	1
	**Fabry disease**	2	9					2	9
	**MLD**	1	29					1	29
	**MPSIH**	1	8					1	8
	**MPSIIIA**	1	1					1	1
	**X-ALD**	2	21					2	21
	**Total**	8	69					8	69
**TOTAL**		34	279	20	118	1	9	55	406

Abbreviations: PID= primary immunodeficiencies; IBMFS= Inherited Bone Marrow Failure Syndromes; pts = patients.

Supplementary Table 3: Distribution of the individual available data within trials for data indicating survival, myeloid engraftment and lymphoid engraftment

Author	Year	Disease	n (survival)	n (myeloid engraftment)	n (lymphoid engraftment)
**Calbi**	2019	MLD	29	27	27
**Aiuti**	2020	ADA-SCID	24	21	21
**Ferrua**	2019	WAS	17	16	16
**Migliavacca**	2020	ADA-SCID	12	11	11
**Braun**	2014	WAS	10	5	3
**Candotti**	2012	ADA-SCID	10	7	
**Gaspar**	2011	X-SCID	10		
**Shaw**	2017	ADA-SCID	10	10	
**Hacein-Bey-**	2014	X-SCID	9		6
**Kohn**	2020	X-CGD	9	7	
**Magnani**	2020	WAS	9	3	4
**Scaramuzza**	2020	β-thalassaemia	9	9	9
**Six**	2020	X-SCID	9		
**Bernardo**	2020	MPSIH	8	6	6
**Mamcarz**	2019	X-SCID	8	6	6
**Gaspar**	2011	ADA-SCID	6		
**De Ravin**	2019	X-SCID	5	2	2
**Esrick**	2019	Sickle cell disease	5		
**Labrosse**	2019	WAS	5		
**Malech**	1997	X-CGD	5		
**Aubourg**	2020	X-ALD	4		
**AvroBio**	2020	Fabry disease	4		
**Magnani**	2020	X-CGD	4		
**Rìo**	2020	Fanconi anemia	4	4	4
**Chinen**	2007	X-SCID	3	3	3
**Kang**	2010	X-CGD	3		3
**Kohn**	2008	ADA-SCID	3		
**Ribeil**	2017	Sickle cell disease	3		
**Adair**	2018	Fanconi anemia	2		
**Cavazzana-Calvo**	2010	β-thalassaemia	2		
**Czechowicz**	2020	Fanconi anemia	2		
**Hyoung Jin Kang**	2011	X-CGD	2		2
**Otsu**	2015	ADA-SCID	2		
**Ott**	2006	X-CGD	2	2	2
**Siler**	2015	X-CGD	2	2	
**Thrasher**	2005	X-SCID	2	2	2
**Barshop**	2020	Cystinosis	1		
**Gaspar**	2006	ADA-SCID	1	1	1
**Kinsella**	2020	MPSIIIA	1		
**Kohn**	2020	LAD-I	1		
**Morris**	2017	WAS	1	1	1
**Six/Ginn**	2010	X-SCID	1		
**Uchiyama**	2019	X-CGD	1		
**Walters**	2019	Sickle cell disease		1	
**Total**			**260**	**146**	**129**

Supplementary Table 4: Details of the 21 deaths

Publication	Disease	Vector	Conditioning	Age at GT (years)	Oncogenic event	HSCT	HSCT after GT (years)	Survival after GT (years)	Details on deaths
Braun, 2014	WAS	γRV	S	12	ALL	Yes	3.5	3.67	ALL relapse shortly after allogeneic HSCT and death
Braun, 2014	WAS	γRV	S	3	ALL	Yes	3.8	3.92	After ALL, patient developed AML during maintenance therapy, haploidentical HSCT, pulmonary insufficiency and hemorrhage then death
Braun, 2014	WAS	γRV	S	-	-	-	-	-	-
Ott, 2006	X-CGD	γRV	S	26	MDS	No	-	2.25	Death for multiorgan failure
Ott, 2006	X-CGD	γRV	S	25	MDS	Yes	3.75	3.75	Death after III HSCT
Six, 2020	X-SCID	γRV	N	0.08	ALL	Yes	3.33	5	Death from T-ALL complications
Six/Ginn, 2010	X-SCID	γRV	N	0.5	No	Yes	2.17	3.67	Death for fungal pneumonia 18 months after allograft
Calbi, 2019	MLD	LV	M	5.67	No	No	-	0.75	Death for disease progression
Calbi, 2019	MLD	LV	M	5.92	No	No	-	0.25	Death for disease progression
Calbi, 2019	MLD	LV	M	0.92	No	No	-	1	Death for ischemic stroke
De Ravin, 2019	X-SCID	LV	S	22	No	No	-	2.25	Death for fatal bronchial bleed due to prior bronchiectasias
Eichler, 2017	X-ALD	LV	M	-	-	No	-	1.8	Death for a viral infection complicated by rhabdomyolysis, acute kidney and liver failure
Eichler, 2017	X-ALD	LV	M	-	-	Yes	-	-	The patient withdrew from the study and later died from complications of allogeneic transplantation
Ferrua, 2019	WAS	LV	S	35	No	No	-	0.2	Death for pre-existing neurodegenerative process
Kanter, 2020	SCD	LV	M	-	No	No	-	1.66	Death for cardiac arrest after sudden shortness of breath
Kohn, 2020	X-CGD	LV	M	4	No	No	-	0.25	Death for a hyperacute idiopathic pneumonitis
Kohn, 2020	X-CGD	LV	M	0.12	No	No	-	0.08	Death for cerebral bleed into pre-existing fungal infection site
Magnani, 2020	WAS	LV	M	10	No	No	-	0.58	Death for opportunistic viral infection
Magnani, 2020	WAS	LV	M	30	No	No	-	4	Death for pneumococcal sepsis and H1N1 influenza
Walters, 2019	SCD	LV	M	41	No	Yes	-	-	Death for HSCT complications. Patient developed MDS after 36 months (non GT-related)
Hacein-Bey-Abina, 2014	X-SCID	SIN- γRV	N	0.75	No	No	-	0.33	Death for adenoviral infection

Abbreviations: N: no conditioning; M: myeloablative; S: submyeloablative/non-myeloablative

Supplementary Table 5: Details of the oncogenic events in 19 patients with individual data available

The two missing events are from the Braun trial (2014).

Publication	Disease	Conditioning	Age at GT (years)	Engraftment failure	Oncogenic event	Onset after GT (years)	Insertion site	HSCT	HSCT after GT (years)	Death	Follow-up (years)
Aiuti, 2020*	ADA-SCID	S	1	No	ALL	4.67	-	No	.	No	4.67
Braun, 2014	WAS	S	3	No	AML	5.08	LMO2; MRPS28; IQSEC2	Yes	7.08	No	7.17
Braun, 2014	WAS	S	12	No	ALL	2.98	C11orf74; TMEM217; LMO2; UBB; TAL1; ST8SIA6; CPSF6; CD46; RIN3	Yes	3.5	Yes	3.67
Braun, 2014	WAS	S	4	No	ALL	1.36	LMO2	Yes	2.25	No	4.25
Braun, 2014	WAS	S	3	No	ALL	3.07	LMO2; TAL1	Yes	3.58	No	3.83
Braun, 2014	WAS	S	3	No	ALL	2.2	LMO2; CYRIP; IMMP2L; GSDMC; TRMT1	Yes	3.83	Yes	3.92
Braun, 2014	WAS	S	2	No	AML	3.24	MECOM	Yes	3.5	No	3.58
Braun, 2014	WAS	S	14	No	ALL	3.79	-	Yes	.	No	3.58
Gaspar, 2011	X-SCID	N	1.08	No	ALL	2	LMO2	No	.	No	4.5
Ott, 2006	X-CGD	S	26	No	MDS	2.25	MECOM	No	.	Yes	2.25
Ott, 2006	X-CGD	S	25	No	MDS	2.33	MECOM	Yes	3.75	Yes	3.75
Siler, 2015	X-CGD	S	4.83	Yes	MDS	3.42	MECOM	Yes	5.48	No	7.25
Siler, 2015	X-CGD	S	8.58	No	MDS	0.67	MECOM/CAMTA1; MECOM/STAT3	Yes	2.63	No	4.8
Six, 2020	X-SCID	N	0.08	No	ALL	2.5	LMO2	Yes	3.33	Yes	5
Six, 2020	X-SCID	N	0.25	No	ALL	2.83	LMO2	No	.	No	14
Six, 2020	X-SCID	N	0.92	No	ALL	5.67	CCND2	No	.	No	13
Six, 2020	X-SCID	N	0.5	No	ALL	14.83	LMO2	No	.	No	14.83
Six, 2020	X-SCID	N	0.75	No	ALL	2.75	LMO2, BMI1	No	.	No	12
Uchiyama, 2019	X-CGD	S	27	Yes	MDS	2.67	MECOM	Yes	.	No	4.1

Abbreviations: No: no conditioning; S: submyeloablative/non-myeloablative.

*https://www.ema.europa.eu/en/medicines/dhpc/strimvelisr-autologous-cd34-enriched-cell-fraction-contains-cd34-cells-transduced-retroviral-vector

Supplementary Table 6: The Newcastle-Ottawa Scale (NOS) for assessing the quality of non-randomised studies in meta-analysis

Note: A study can be awarded with a score for each numbered item within the Selection and Outcome domains. A maximum of 15 points can be assigned for each study.

**SELECTION**	**Score**				
	**3**	**2**	**1**	**0**	
**1) Representativeness of the exposed cohort**	Truly representative of the average of patients potentially requiring a treatment with gene therapy in the community	Somewhat representative of the average patients potentially requiring a treatment with gene therapy in the community	Selected group	No description of the derivation of the cohort	
**2) Ascertainment of exposure GT**	Secure record	Structured interview	Written self-report	No description	
**3) Demonstration that outcome of interest was not present at start of study (oncogenic event)**			Yes	No	
**OUTCOME**	**Score**				
	**4**	**3**	**2**	**1**	**0**
**1) Assessment of outcome**		Independent blind assessment	Record linkage	Self-report	No description
**2) Was the follow-up of each study long enough for outcomes to occur?**				Yes (>2 years of follow-up)	No
**3) Adequacy of follow-up of cohorts**	Complete follow up: all subjects accounted for	Subjects lost to follow up (20%) were unlikely to introduce bias because small numbers were lost: >80 % had follow up, or description was provided of those lost	Follow up rate < 80% and there was no description of those lost	No statement	
