# Supplementary material for: A systematic review and meta-analysis of gene therapy with hematopoietic stem and progenitor cells for monogenic disorders
Source: Nat Commun. 2022 Mar 14;13:1315. doi: 10.1038/s41467-022-28762-2 (PMC8921234; doi:10.1038/s41467-022-28762-2)
Supplement: Supplementary file 2 — Reporting Summary [file 41467_2022_28762_MOESM2_ESM.pdf]

nature portfolio

Corresponding author(s): Alessandro Aiuti

Last updated by author(s): Nov 15, 2021

Reporting Summary

Nature Portfolio wishes to improve the reproducibility of the work that we publish. This form provides structure for consistency and transparency in reporting. For further information on Nature Portfolio policies, see our Editorial Policies and the Editorial Policy Checklist.

Statistics

For all statistical analyses, confirm that the following items are present in the figure legend, table legend, main text, or Methods section.

n/a	Confirmed	
☐	☒	The exact sample size (n) for each experimental group/condition, given as a discrete number and unit of measurement
☐	☒	A statement on whether measurements were taken from distinct samples or whether the same sample was measured repeatedly
☐	☒	The statistical test(s) used AND whether they are one- or two-sided<br>*Only common tests should be described solely by name; describe more complex techniques in the Methods section.*
☐	☒	A description of all covariates tested
☐	☒	A description of any assumptions or corrections, such as tests of normality and adjustment for multiple comparisons
☐	☒	A full description of the statistical parameters including central tendency (e.g. means) or other basic estimates (e.g. regression coefficient) AND variation (e.g. standard deviation) or associated estimates of uncertainty (e.g. confidence intervals)
☐	☒	For null hypothesis testing, the test statistic (e.g. F, t, r) with confidence intervals, effect sizes, degrees of freedom and P value noted<br>*Give P values as exact values whenever suitable.*
☐	☒	For Bayesian analysis, information on the choice of priors and Markov chain Monte Carlo settings
☐	☒	For hierarchical and complex designs, identification of the appropriate level for tests and full reporting of outcomes
☐	☒	Estimates of effect sizes (e.g. Cohen's d, Pearson's r), indicating how they were calculated

Our web collection on statistics for biologists contains articles on many of the points above.

Software and code

Policy information about availability of computer code

Data collection	We extracted aggregated (and when possible individual data) from the pubblications included in the meta-analysis and we stored them in a excel file (Excel 2016)
Data analysis	We used the software R (version 3.6) and the package metafor specifically for meta-analysis.

For manuscripts utilizing custom algorithms or software that are central to the research but not yet described in published literature, software must be made available to editors and reviewers. We strongly encourage code deposition in a community repository (e.g. GitHub). See the Nature Portfolio guidelines for submitting code & software for further information.

Data

Policy information about availability of data

All manuscripts must include a data availability statement. This statement should provide the following information, where applicable:

- Accession codes, unique identifiers, or web links for publicly available datasets
- A description of any restrictions on data availability
- For clinical datasets or third party data, please ensure that the statement adheres to our policy

Because this meta-analysis was based on data extracted from previously published research, most of the data and study materials are available in the public domain. Data from this additional analysis will not be made publicly available; however, we encourage interested parties to contact the corresponding author for further discussions.

Field-specific reporting

Please select the one below that is the best fit for your research. If you are not sure, read the appropriate sections before making your selection.

- [x] Life sciences
- [ ] Behavioural & social sciences
- [ ] Ecological, evolutionary & environmental sciences

For a reference copy of the document with all sections, see nature.com/documents/nr-reporting-summary-flat.pdf

Life sciences study design

All studies must disclose on these points even when the disclosure is negative.

Sample size	Not applicable.
Data exclusions	To be eligible, studies must have: (1) included patients affected by monogenic inherited diseases treated with HSPCS-GT; (2) reported outcomes, including numbers of deaths, genotoxicities and engraftments. Genotoxic events were intended as the first occurred haematological malignancy related or probably related to GT. Second malignancies and tumors not related to GT were not included in the genotoxicity analysis. Engraftment was considered successful when molecular tests reported the presence of gene corrected cells for ≥1 year post-GT.
Replication	Our study is a Meta-analysis and we mainly deal with aggregated data extracted from various pubblications
Randomization	Not applicable. The trials included in our meta-analysis refer to phase I/II studies of an innovative treatment in the context of rare diseases where only one arm studies are conducted.
Blinding	The blinding we applied was 1) in reviewing the abstracts of articles obtained from the search and 2) in evaluating the methodological quality of the studies included in the meta-analysis. These two steps were done in a blinded fashion by 2 authors (AA and FT).

Reporting for specific materials, systems and methods

We require information from authors about some types of materials, experimental systems and methods used in many studies. Here, indicate whether each material, system or method listed is relevant to your study. If you are not sure if a list item applies to your research, read the appropriate section before selecting a response.

Materials & experimental systems

n/a	Involved in the study	
x		Antibodies
x		Eukaryotic cell lines
		Palaeontology and archaeology
x		Animals and other organisms
	x	Human research participants
	x	Clinical data
x		Dual use research of concern

Methods

n/a	Involved in the study	
x		ChIP-seq
x		Flow cytometry
x		MRI-based neuroimaging

Palaeontology and Archaeology

Specimen provenance	*Provide provenance information for specimens and describe permits that were obtained for the work (including the name of the issuing authority, the date of issue, and any identifying information). Permits should encompass collection and, where applicable, export.*
Specimen deposition	*Indicate where the specimens have been deposited to permit free access by other researchers.*
Dating methods	*If new dates are provided, describe how they were obtained (e.g. collection, storage, sample pretreatment and measurement), where they were obtained (i.e. lab name), the calibration program and the protocol for quality assurance OR state that no new dates are provided.*

- [ ] Tick this box to confirm that the raw and calibrated dates are available in the paper or in Supplementary Information.

Ethics oversight	*Identify the organization(s) that approved or provided guidance on the study protocol, OR state that no ethical approval or guidance was required and explain why not.*

Note that full information on the approval of the study protocol must also be provided in the manuscript.

Human research participants

Policy information about studies involving human research participants

Population characteristics	*Describe the covariate-relevant population characteristics of the human research participants (e.g. age, gender, genotypic information, past and current diagnosis and treatment categories). If you filled out the behavioural & social sciences study design questions and have nothing to add here, write "See above."*
Recruitment	*Describe how participants were recruited. Outline any potential self-selection bias or other biases that may be present and how these are likely to impact results.*
Ethics oversight	*Identify the organization(s) that approved the study protocol.*

Note that full information on the approval of the study protocol must also be provided in the manuscript.

Clinical data

Policy information about clinical studies

All manuscripts should comply with the ICMJEguidelines for publication of clinical research and a completedCONSORT checklist must be included with all submissions.

Clinical trial registration	*Provide the trial registration number from ClinicalTrials.gov or an equivalent agency.*
Study protocol	*Note where the full trial protocol can be accessed OR if not available, explain why.*
Data collection	Searches were conducted in PubMed, Embase and Cochrane Central Register of Controlled Trials to identify potentially eligible literature from inception to October 2020.
Outcomes	*Describe how you pre-defined primary and secondary outcome measures and how you assessed these measures.*
